# Over/Undervoltage and Undervoltage Shift of Hybrid Islanding Detection Method of Distributed Generation

**DOI:** 10.1155/2015/654942

**Published:** 2015-03-23

**Authors:** Manop Yingram, Suttichai Premrudeepreechacharn

**Affiliations:** Department of Electrical Engineering, Faculty of Engineering, Chiang Mai University, Chiang Mai 50200, Thailand

## Abstract

The mainly used local islanding detection methods may be classified as active and passive methods. Passive methods do not perturb the system but they have larger nondetection zones, whereas active methods have smaller nondetection zones but they perturb the system. In this paper, a new hybrid method is proposed to solve this problem. An over/undervoltage (passive method) has been used to initiate an undervoltage shift (active method), which changes the undervoltage shift of inverter, when the passive method cannot have a clear discrimination between islanding and other events in the system. Simulation results on MATLAB/SIMULINK show that over/undervoltage and undervoltage shifts of hybrid islanding detection method are very effective because they can determine anti-islanding condition very fast. Δ*P*/*P* > 38.41% could determine anti-islanding condition within 0.04 s; Δ*P*/*P* < −24.39% could determine anti-islanding condition within 0.04 s; −24.39% ≤ Δ*P*/*P* ≤ 38.41% could determine anti-islanding condition within 0.08 s. This method perturbed the system, only in the case of −24.39% ≤ Δ*P*/*P* ≤ 38.41% at which the control system of inverter injected a signal of undervoltage shift as necessary to check if the occurrence condition was an islanding condition or not.

## 1. Introduction

The Distributed generation (DG) connected to the power system which they are more and more popular, especially small DGs derived from renewable energy [1]. Features of DG include security of electricity supply to customers, liberalization of the electricity market, reduced CO_2_ emission by the introduction of renewable energy sources, increased power availability and reliability, increased standby capacity, improved power quality, grid support, combined generation of heat and power, and cost saving of adding more remote generating sources [2]. However, the advent of DG makes some problems to the stability and the power quality in the adjacent utility. Specially, most issued problem is islanding phenomenon.

Islanding is a condition in which a portion of the utility system that contains both load and generation is isolated from the remainder of the utility system. Phenomena of islanding condition may occur for several reasons such a result of a fault that is detected by the utility but is not detected by the DG, a result of an accidental opening of the normal utility supply by an equipment failure, a result of human error or malicious mischief, an act of nature, and so forth. Cause of islanding condition should be avoided because the utility cannot control voltage and frequency in the island at which there is the possibility of damage to equipment because of voltage or frequency excursions outside of the acceptable ranges on which the utility has no control and so forth [3, 4].

Islanding detection techniques can be divided into local and remote techniques. The local techniques can further be divided into passive, active, and hybrid techniques. Remote islanding detection techniques are follows these detection techniques are based on some kind of communication between the grid and the DG. They are more reliable than the local techniques, but they are more expensive to implement. Local islanding detection techniques are based on the measurement of some parameters (voltage, current, and frequency, among others) on the distributed generator side. They are classified as passive, based exclusively on the monitoring of these parameters, and active techniques, which intentionally introduce disturbances at the output of the inverter and observe whether the parameters outlined above are affected. Hybrid methods employ both the active and passive detection techniques [5].

Local islanding detection techniques are interested in research and develop because they are suitable for small DGs (small DGs are less than or equal to 10 kW [3]), as remote islanding detection techniques are more expensive than local islanding detection techniques and trends of local islanding detection techniques used in small DGs are active islanding detection. However, due to the increasing trend of small DGs continuously, they will increasingly deteriorate the quality of grid voltage as well. So, this paper proposed over/undervoltage and undervoltage shift of hybrid islanding detection method of distributed generation to reduce deteriorating the quality of grid voltage when compared with the active islanding detection. In general development anti-islanding detection technique should accomplish the following goals: detecting islanding rapidly enough to guarantee personnel and equipment safety and safeguard the reliability and integrity of electric power system and DG systems, disconnecting the DG system only when islanding is actually occurring, minimal hardware requirements, and requiring minimal or no interaction with normal electric power system operation and control [6]. The goals especially must have relevant standards including IEEE Std. 929, IEEE Std. 1547, the international standard IEC 62116, UL 1741, Japanese Standard (JET Std. 2002), and Korean Standard (Korean PV 501, 2008) which the key point of all the standards are same or similar conclusion that the DG interconnection system shall detect the island and cease to energize the electric power systems network within two seconds of the formation of an island when islanding condition occurs [7, 8]. In this paper, we used values in IEC 62116 because they consistent with the electric power system in Thailand AC 220 volts 50 Hz.

Presentation of a new hybrid islanding detection technique includes nondetection zone of over/undervoltage of inverter-based DG showing finding nondetection zone and showing the relationship between over/undervoltage and Δ*P*/*P* after islanding condition occurs; the relationship between load and NDZ of OUV of inverter-based DG shows the change of load effect to enter or leave the NDZ; the relationship between voltage and NDZ of OUV of inverter-based DG shows phenomenon of overvoltage or undervoltage in the inverter; proposed methodology shows over/undervoltage and undervoltage shift islanding detection algorithm, demonstrating the methodology on MATLAB/SIMULINK which proves the new methodology by simulation and conclusion. The new hybrid method was used, an over/undervoltage has been used to initiate an undervoltage shift, when the over/undervoltage method cannot have a clear discrimination between islanding and other events in the system. Simulation results show that the new hybrid method is very effective because it can determine anti-islanding condition very fast.

## 2. NDZ of OUV of Inverter-Based DG

In this paper, over/undervoltage (OUV) method was used to initiate the islanding detection because it is highly effective in group of passive islanding detection methods. However, the OUV method has a weakness because it has a wide nondetection zone. This section analyzed the NDZ of the OUV technique. “Nondetection zone” (NDZ) can be defined as the range in terms of the difference between the power supplied by the DG inverter and that consumed by the load, in which an islanding detection scheme under test fails to detect this condition [9].

**Figure 1:**
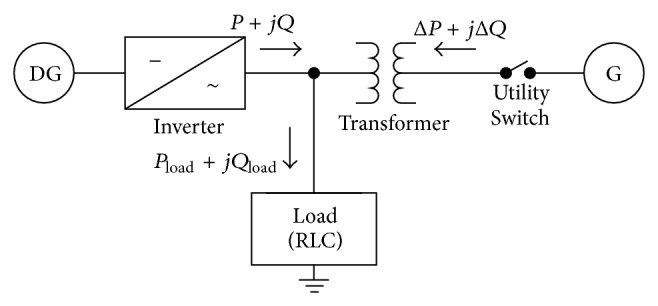
Power balance of the system.

The over/undervoltage technique allows detection of the islanding phenomenon through the measure of voltage at the point of common coupling (PCC) and subsequent comparison with the limits set proper operation. If the measured values are outside the established range, the inverter is stopped or disconnected.  Figure 1 shows the power balance of the system [9].

**Figure 2:**
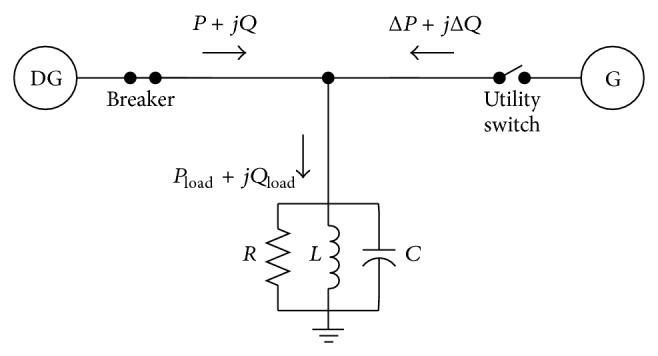
DG system configuration and power flows.

The OUV technique received the nondetection zone by analysis. To facilitate this analysis, Figure 1 was improved to be Figure 2 [8].

**Figure 3:**
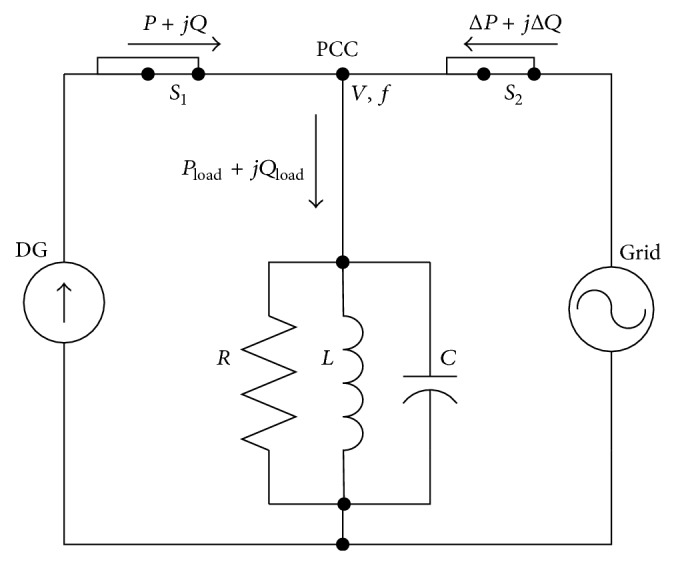
Equivalent circuit of grid-connected DG power generation system.

It is usually assumed that the local load can be modeled as a parallel RLC circuit because, for most islanding detection methods (IDM), some types of RLC loads cause the most difficulty in detection. The equivalent circuit of the grid connected to the DG power generation system is shown in Figure 3 [8, 10]. Power flows show, in Figure 3, node “PCC” is the PCC between the utility grid and DG system. The utility grid voltage source at the right can be disconnected from node “PCC” by the switch S_2_ (breaker/recloser). A local load is also connected at the PCC.

When the utility grid is connected (breaker is closed), the active and reactive power *P* + *jQ* flows from the DG system to node “PCC” and *P*_load_ + *jQ*_load_ flows from node “PCC” to the local load. The power flows from the utility grid to node “PCC” are Δ*P* + *j*Δ*Q*.

These power equations are shown in

(1) $$\begin{array}{c} P_{\text{load}}=P+\Delta P,\\ Q_{\text{load}}=Q+\Delta Q. \end{array}$$

The amplitude and phase angle of RLC parallel load impedance, resonant frequency *f*_0_, and quality factor *Q*_*f*_ are defined in

(2) $$\begin{array}{c} \left|z\right|=\frac{1}{\sqrt{1/R^{2}+{\left(1/\omega L-\omega C\right)}^{2}}},\\ \phi_{\text{load}}=\text{ta}{\text{n}}^{-1}\left[Q_{f}\left(\frac{f_{0}}{f}-\frac{f}{f_{0}}\right)\right],\\ f_{0}=\frac{1}{2\pi \sqrt{LC}},\\ Q_{f}=R\sqrt{\frac{C}{L}}. \end{array}$$

The nondetection zone of active power (nondetection zone of over/undervoltage) is

(3) $$\begin{array}{c} {\left(\frac{V}{V_{\max}}\right)}^{2}-1\leq \frac{\Delta P}{P}\leq {\left(\frac{V}{V_{\min}}\right)}^{2}-1. \end{array}$$

From IEC 62116 set *V*_max⁡_ = 115%, *V*_min⁡_ = 85%  (3) [7],

(4) $$\begin{array}{c} -24.39\%\leq \frac{\Delta P}{P}\leq 38.41\%. \end{array}$$

**Figure 4:**
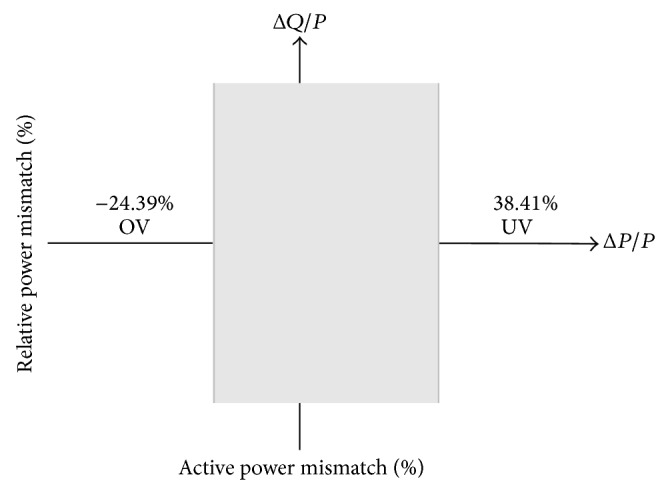
Nondetection zone of over/undervoltage.

Therefore, the NDZ of OUV is shown in Figure 4.

## 3. The Relationship between Load and NDZ of OUV of Inverter-Based DG

Analysis in topic 2 showed that the OUV technique had a wide nondetection zone. When NDZ was wide, failure of islanding detection increased as well. This section shows that variation of load always occurs in real systems. The variation of load can affect Δ*P*/*P*, at which Δ*P*/*P* can enter into NDZ of OUV shown in Figure 4. Wherewith, scale of NDZ of OUV is the percentage of Δ*P*/*P*; it is possible to use a simulator program with scale applications in MW instead of changing load with scale in kW or less. Therefore, this section used Powerworld Simulator Program for the proposed relationship between load and NDZ of OUV.

**Figure 5:**
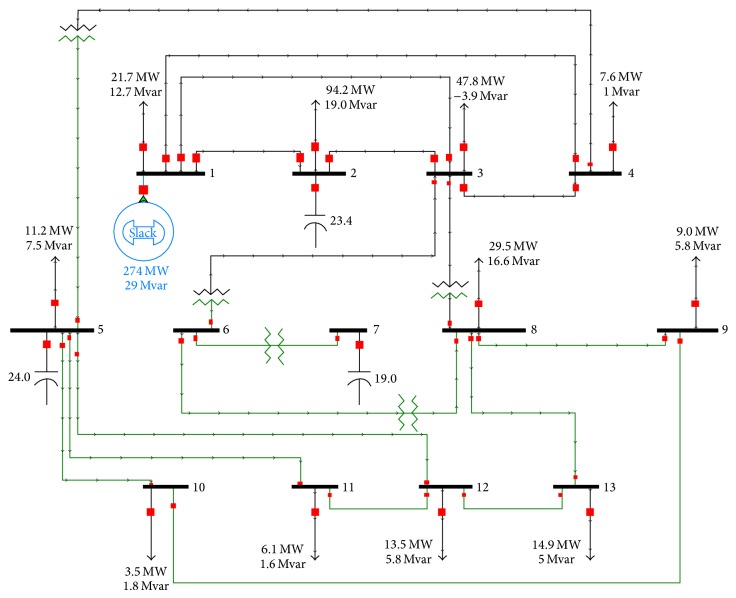
The 13- bus system for simulation.

The data used in the analysis of the IEEE 14-bus system was based on system data of the Provincial Electricity Authority (PEA) of Thailand. Some of the data are modified and converted to the format, and the per-unit for the data was used in Powerworld Simulator Program Version 16. The data entered into the program is a circuit of Figure 5. The parameters of system are also show in Tables 1 and 2. As for the results of simulation, generator 1 (as main generator) supplied power to the load demand 274 MW and 29 MVar.

**Figure 6:**
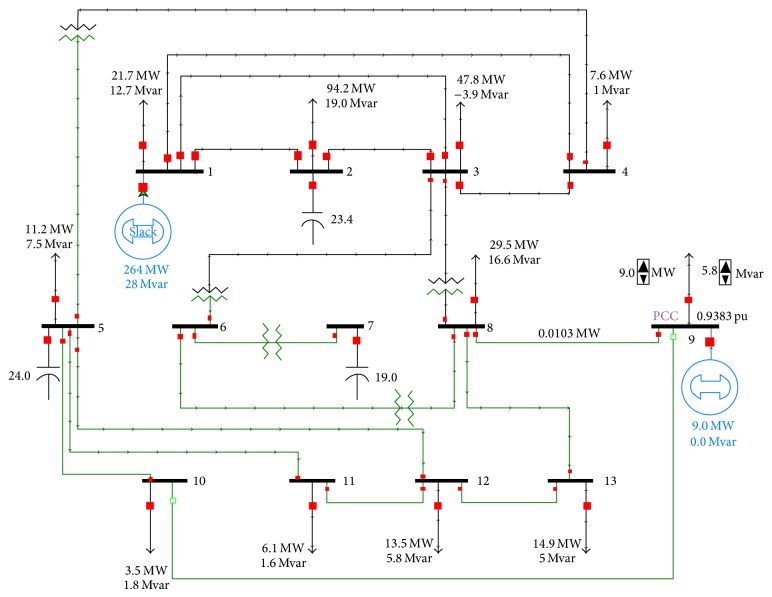
DG in 13-bus system.

**Figure 7:**
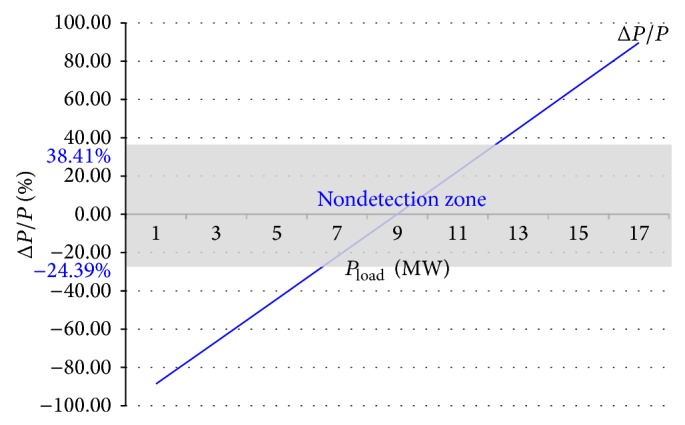
The changes of load affect entering or leaving the NDZ.

The 13-bus system has added DG type PV size 9 MW at bus 9. For easy analysis, off power flow of a transmission line connected bus 10 and bus 9 as shown in Figure 6. After that, size of load (fixed PF = 0.84 lagging) was adjusted, and data was recorded into Table 3, which is plotted in the graph shown in Figure 7.

From Table 3 and Figure 7, it is shown that variation of load, which always occurs in real systems, directly affects the OUV islanding detection method because the variation of load makes uneven Δ*P* when uneven Δ*P* directly affects uneven Δ*P*/*P*. Consider Figure 4; it is seen that the uneven Δ*P*/*P*, the Δ*P*/*P* enter nondetection zone at some time. If Δ*P*/*P* enters the nondetection zone when islanding condition occurs, voltage at PCC (*V*_PCC_) will not be less than undervoltage and will not be more than overvoltage. This phenomena are showed in the next section [11].

## 4. The Relationship between Voltage and NDZ of OUV of Inverter-Based DG

Section 3 showed variation of load, which always occurs in real systems. They can affect Δ*P*/*P* into NDZ of OUV. This section presents voltage at PCC (*V*_PCC_) after islanding condition occurs while Δ*P*/*P* enters or leaves the NDZ. Experiments use a real inverter.

The experiment was the same as the anti-islanding testing diagram defined in UL 1741-1999, IEEE Std. 929-2000 and IEEE Std. 1547-2003 [3, 4, 8]. There is a specific definition for RLC load as a testing condition. The resonant frequency of the RLC load is the same as grid line frequency. Usually the unity power factor condition, combined with the RLC load, is the worst case for islanding detection when the active power or the reactive power is 100% matched between the load and the DG output [3, 8]. The experiment built islanding condition by off-grid (off switch) between the point of common coupling (PCC) and utility. Experimental results were recorded after testing reliability and accuracy [12].

**Figure 8:**
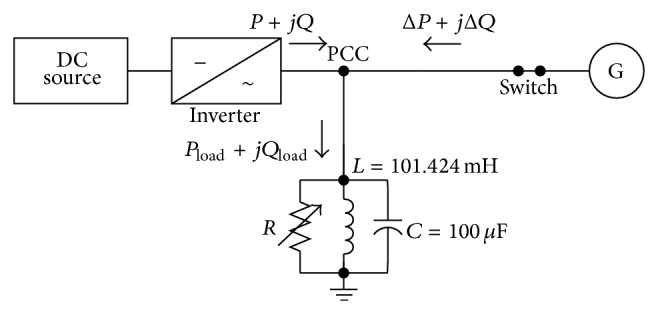
Experimental circuit diagram by use of inverter.

**Figure 9:**
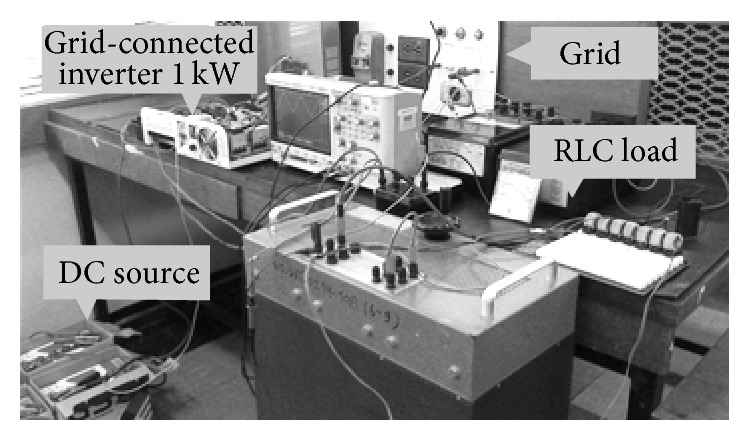
Experiment in research and developing of power electronic laboratory.

The experiment shows the relationship between voltage and NDZ of OUV by use of inverter as shown in Figures 8 and 9.

In Figure 8 the experimental circuit diagram *X*_*L*_ = 31.48 Ohm and *X*_*C*_ = 31.48 Ohm. The load is resonant condition because *X*_*L*_ = *X*_*C*_ and this resonant condition makes *Q*_load_ = 0. In the experiment, we have set the following: active power of DG is *P* = 1 kW, power factor of DG is PF = 1, power factor of load is PF_load_ = 1, and active power of load changed from 600 W to 1,200 W (100 W per step). As for the worst case of islanding detection in the experiment, they should happen when active power of load is 1,000 W because active power of load is as valuable as active power of DG.

**Figure 10:**
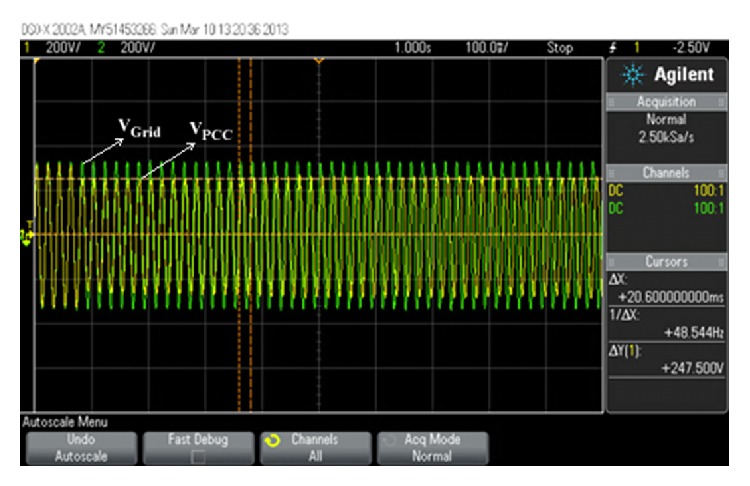
Experimental results of inverter while *P* = 1 kW and *P*_load_ = 1 kW.

The experimental results in Table 4 show *V*_PCC_ (voltage at the point of common coupling), before and after islanding condition happened, which can be seen in column 8 is off-grid voltage; *V*_PCC_ decreases when Δ*P* increases. This result accords with Figure 4, when Δ*P*/*P* increases from negative to positive; it affects off-grid voltage at *V*_PCC_ and decreases from overvoltage to undervoltage when is in comparison with on-grid voltage at *V*_PCC_ (*V*_Grid_ is the same as on-grid voltage at *V*_PCC_). The results show that a nondetection zone (NDZ) of OUV of inverter-based DG comes true [8]. Moreover, if inverters use the over/undervoltage (OUV) technique for anti-islanding when consider in Table 4 seen that the inverter can detect islanding condition in row 4 to row 7 because off-grid voltage at *V*_PCC_ is less than on-grid voltage at *V*_PCC_ multiply 0.85. As is wished, IEC 62116, normal voltage range is 85% ≤ *V* ≤ 115%, if *V* (*V* is the same as on-grid voltage at *V*_PCC_) was more than 115% or less than 85% that shows that islanding occurs and the control system of inverter will cease to energize the load. If the inverter set over/undervoltage of relays at the constant normal voltage of utility is 220 V, overvoltage of relay = 253 V and undervoltage of relay = 187 V. In Table 4, it is seen that the inverter could not detect islanding in only rows 2, 3, and 4 because off-grid voltage at *V*_PCC_ was not more than 253 V or less than 187 V.  Figure 10 shows some figures of the experimental results.

Nevertheless, there are some observations. Firstly, undervoltage of off-grid voltage at *V*_PCC_ happened before Δ*P*/*P* into positive which can be seen at row 4 in Table 4 when *P*_load_ = 900 W, *P* = 1,000 W, and Δ*P*/*P* = −10. *V*_PCC_ before islanding condition was 229.8 V and off-grid voltage at *V*_PCC_ was 191.0 V. Second, normal worst case range is *P*_load_ = *P* at which on-grid voltage at *V*_PCC_ should be similar to off-grid voltage at *V*_PCC_ but the experimental results show that *V*_PCC_ between on-grid voltage at *V*_PCC_ and off-grid voltage at *V*_PCC_ was not similar. Table 4 shows that *V*_PCC_ before and after islanding condition was most similar at *P*_load_ = 800 W, *P* = 1,000 W, and Δ*P*/*P* = −20 at row 3. On-grid voltage at *V*_PCC_ was 231.6 V, and off-grid voltage at *V*_PCC_ was 240.5 V.

However, these experiments are tested as a parallel RLC circuit because for most islanding detection method some type of RLC load causes the most difficulty in detection. Therefore, investigation of the relationship between voltage and NDZ of OUV of local islanding detection technique by use of inverter-based DG [8, 13] is

(5) $$\begin{array}{c} {\left(\frac{V}{V_{\max}}\right)}^{2}-1\leq \frac{\Delta P}{P}\leq {\left(\frac{V}{V_{\min}}\right)}^{2}-1, \end{array}$$

which is consistent with the experimental results.

**Figure 11:**
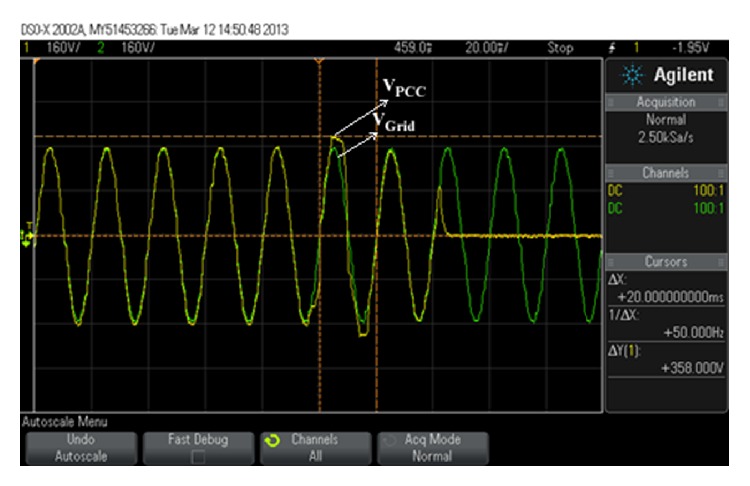
Speed of over/undervoltage islanding detection method.

Figure 11 shows speed of over/undervoltage islanding detection method by inverter, demonstrated on *P*_load_ = 600 W, *P* = 1.000 W, which makes Δ*P*/*P* outside nondetection zone. When islanding condition occurs, the inverter could detect the island and cease to energize the electric power system network within three cycles. As a result, the inverter set over/undervoltage of relays as constant normal voltage of utility was 220 V, overvoltage of relay was 253 V, and undervoltage of relay was 187 V. In Table 4, it can be seen that this case had overvoltage at *V*_PCC_ = 253.2 V.

## 5. Proposed Methodology

**Figure 12:**
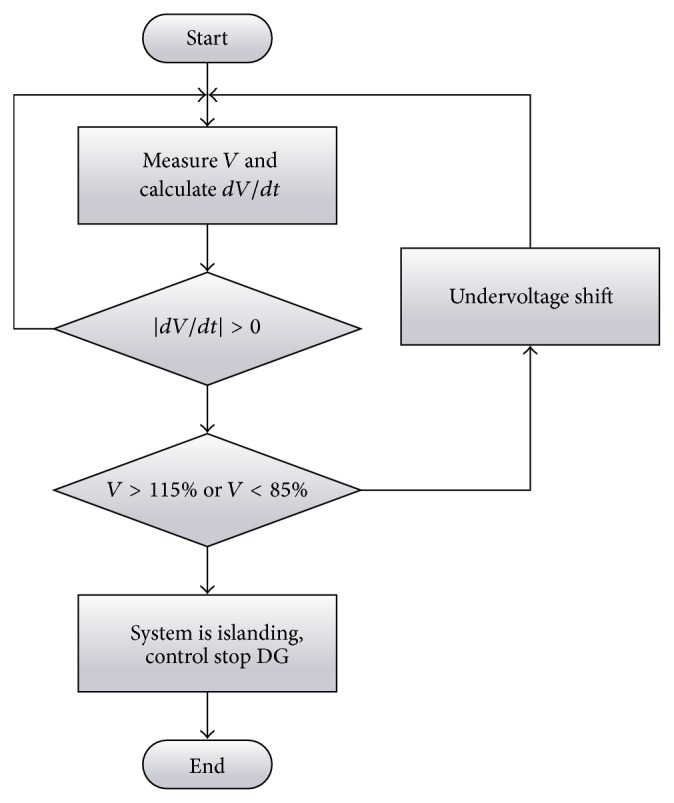
Over/undervoltage and undervoltage shift algorithm.

From the experimental results in Figure 11, it is shown that over/undervoltage of passive islanding detection method has high efficiency because it can detect the island and cease to energize within three cycles, but it has weakness because if *P*_load_ is near *P*, it will cause Δ*P*/*P* to be inside the nondetection zone. When islanding condition occurs, it cannot detect islanding condition because voltage at *V*_PCC_ cannot be more than overvoltage or less than undervoltage, which can be seen from the above presentation. This paper proposes a hybrid islanding detection technique that the algorithm of solving problem had described the flow of the proposed methodology shown in Figure 12.

The beginning of the algorithm detected islanding condition for anti-islanding condition. First, voltage is measured every period at PCC (*V*_PCC_); then *dV*/*dt* of *V*_PCC_ is calculated.

Next, *dV*/*dt* is checked. If |*dV*/*dt* | = 0, this shows that voltage has not changed, and the algorithm will start the process from the beginning. If |*dV*/*dt* | >0, this shows that voltage has changed and the algorithm will do the next process. (In practical systems, the value of |*dV*/*dt*| should be slightly greater than zero. Furthermore, *dF*/*dt* can replace *dV*/*dt*.)

When |*dV*/*dt* | >0, the algorithm compares *V*_PCC_ after islanding condition occurs and over/undervoltage; mean *V*_PCC_ (*V*_PCC_ after islanding condition occurs) is more than 115%∗*V*_PCC_ (*V*_PCC_ before islanding condition occurs) = 253 V or less than 85%∗*V*_PCC_ = 187 V. If it has more than 253 V or less than 187 V, the islanding condition occurs at which the algorithm will cease to energize to the load. If it is not more than 253 V or less than 187 V, the algorithm does the next process.

**Figure 13:**
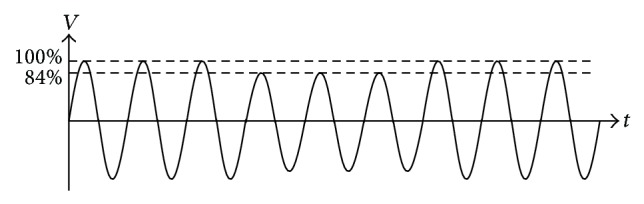
Characteristic of waveform injection into the system.

This process will control the inverter to energize constant voltage at 84% of normal voltage, or 184.8 V (84%∗220 V) to power system. Variation of constant voltage at 84% is a new method in group of active islanding detection techniques. Normal voltage outputs of inverters can be changed, which changes the DC voltage to input of inverter or the gain of the inverter. This paper changes the DC voltage to input of inverter for the AC voltage output of the inverter less than or equal to 84%, So 184.8 V three cycles are injected into the system as shown in Figure 13.

Afterwards, the inverter injected a constant voltage of 184.8 V for three cycles into the system. If this occurrence condition is an islanding condition, the voltage is measured at *V*_PCC_. The *V*_PCC_ will same constant voltage (184.8 V) for three cycles. If *V*_PCC_ has less than 187 V, the algorithm will cease to energize the load. If the occurrence condition is not an islanding condition, *V*_PCC_ will remain the same, but *P* (active power of DG) will decrease inversely with Δ*P* (active power of grid) increasing that can prove the following.

In normal conditions, the active power of load affects the active power of DG and the active power of grid in the point of common coupling. It is possible to calculate the active power variation versus the voltage variation injected in the load [8, 9, 14, 15]. Consider

(6) $$\begin{array}{c} P_{\text{load}}=P_{\text{DG}}+P_{\text{Grid}}=\frac{V^{2}}{R}, \end{array}$$

(7) $$\begin{array}{c} V=\sqrt{R\cdot \left(P_{\text{DG}}+P_{\text{Grid}}\right)}. \end{array}$$

Deriving *P*_DG_ + *P*_Grid_ and from (6)

(8)  ∂(PDG+PGrid)∂V=2·VR, ∂(PDG+PGrid)∂V=2·R·PDG+PGridR, ∂PDG+PGrid∂V=2·PDG+PGridR.

The active power variation is expressed by

(9) $$\begin{array}{c} \Delta P_{\text{DG}}+\Delta P_{\text{Grid}}=2\cdot \Delta V\cdot \sqrt{\frac{P_{\text{load}}}{R}}. \end{array}$$

From (9) it can be seen that *R* and *P*_load_ are constant. When the control system of the inverter injected constant voltage at 184.8 V (84%) for three cycles into the system after synchronizing the DG to the grid at which the occurrence condition is not islanding condition *V*_PCC_ (Δ*V*) remains the same but Δ*P*_DG_ decrease inversely with Δ*P*_Grid_ increases. The changes between active power of DG and active power of grid are determined to be sufficient for the load. This paper shows the changing of active power in the next section.

## 6. Demonstrating the Methodology on MATLAB/SIMULINK

**Figure 14:**
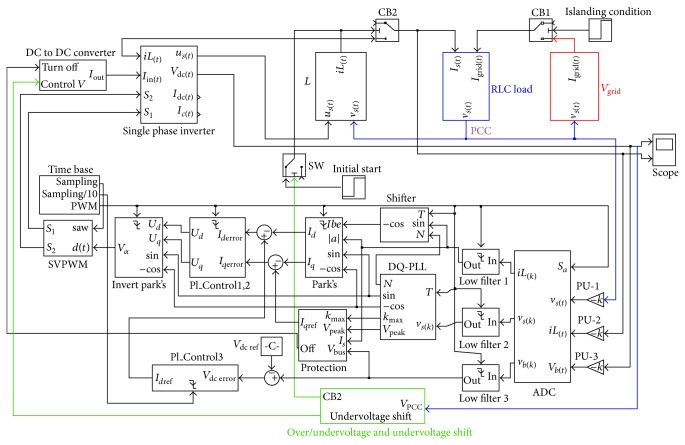
Model of a single phase grid-connected inverter.

**Figure 15:**
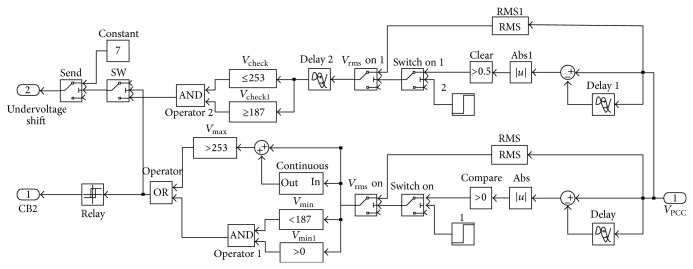
Over/undervoltage and undervoltage shift block.

This section proves the over/undervoltage and undervoltage shift islanding detection algorithm for anti-islanding condition that this algorithm may need to develop in the future. This algorithm uses MATLAB/SIMULINK for the presentation. The model used in the demonstration, as in Figure 14, tests with a parallel constant RLC load and resonant frequency of the constant RLC load that is the same as grid line frequency. In Figure 15 is over/undervoltage and undervoltage shift block from the model of Figure 14.

In the simulation to propose over/undervoltage and undervoltage shift block of hybrid islanding detection method, every case was a presentation with reference in Figure 4 which shows the relationship between Δ*P*/*P* and voltage after islanding condition occurs. The presentations include the case of Δ*P*/*P* > 38.41%, case of Δ*P*/*P* < −24.39%, and case of −24.39% ≤ Δ*P*/*P* ≤ 38.41%. In addition, this paper shows the case of injecting a signal to change the undervoltage but the occurrence condition is not an islanding condition.

### 6.1. Case of Δ*P*/*P* > 38.41%

**Figure 16:**
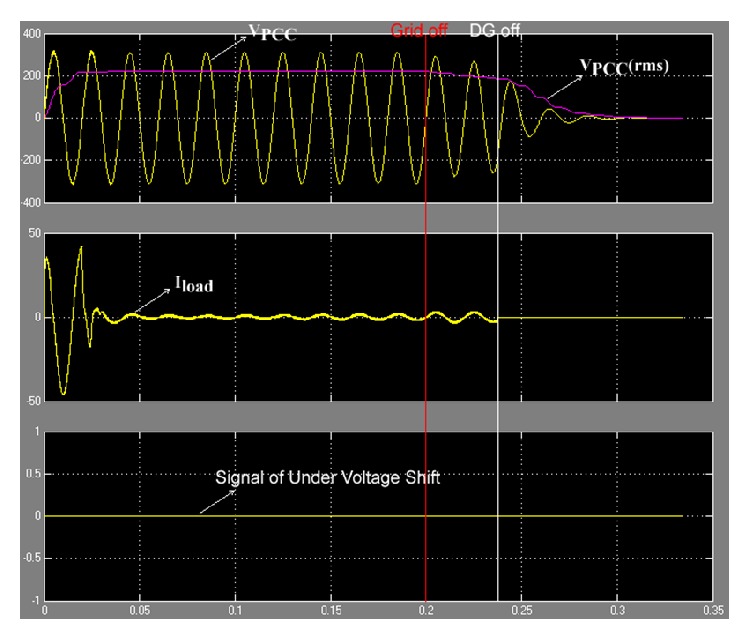
*V*_PCC_ on undervoltage.

This case will happen when *P* < *P*_load_ by enough to make  Δ*P*/*P* > 38.41%. When islanding condition occurs, *V*_PCC_ will be less than 187 V (undervoltage of *V*_PCC_). The proposed method was proved by simulation on MATLAB/SIMULINK, and the model was set for islanding condition at 0.2 s. The results of simulation are show in Figure 16.

From Figure 16 it can be seen that when the grid was off, islanding condition at 0.2 s, *V*_PCC_ decreased steadily. The control system of the inverter ordered DG off at the point of *V*_PCC_  (rms) < 187 V. In Figure 16 it can be seen that the DG ceased to energize the electric power systems network at 0.24 s, showing that the proposed method used times for the anti-islanding condition of case of Δ*P*/*P* > 38.41% were within 0.04 s.

### 6.2. Case of Δ*P*/*P* < −24.39%

**Figure 17:**
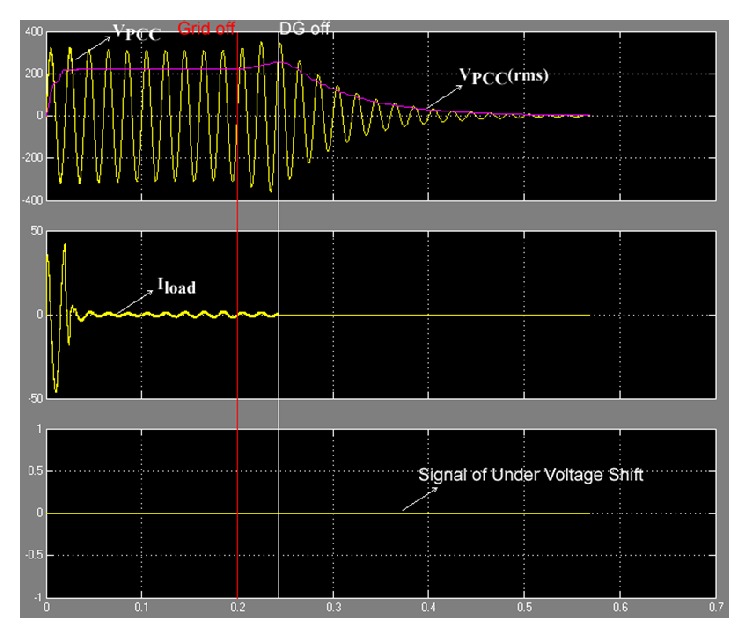
*V*_PCC_ on overvoltage.

This case will happen when *P* > *P*_load_ by enough to make  Δ*P*/*P* < −24.39%. When an islanding condition occurs, *V*_PCC_ will be more than 253 V (overvoltage of *V*_PCC_). The model set the islanding condition at 0.2 s, which the simulation result show in Figure 17.

From Figure 17 it can be seen that when the grid was off, islanding condition at 0.2 s, *V*_PCC_ increased steadily. The control system of the inverter ordered DG off at the point of *V*_PCC_  (rms) > 253 V. In Figure 17 it can be seen that the DG ceased to energize the electric power systems network at 0.24 s, showing that the proposed method used times for the anti-islanding condition of case of Δ*P*/*P* < −24.39% were within 0.04 s.

### 6.3. Case of −24.39% ≤ Δ*P*/*P* ≤ 38.41%

**Figure 18:**
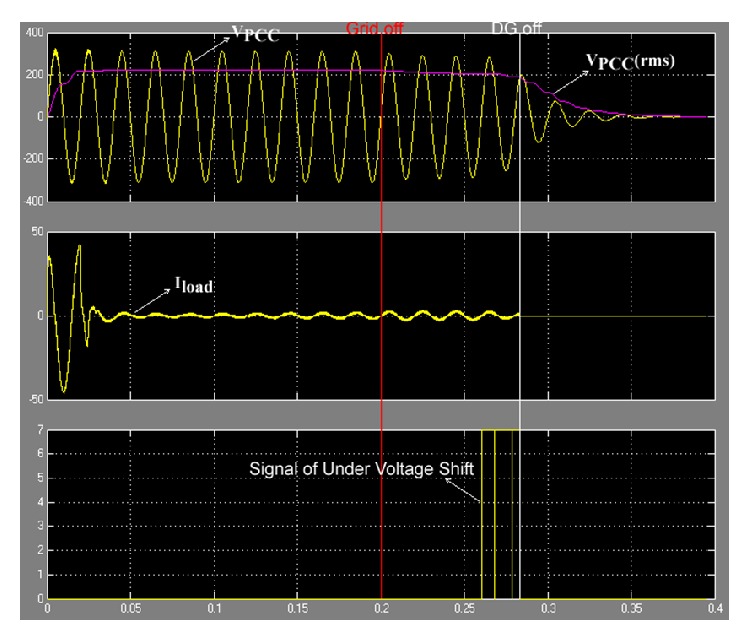
*V*_PCC_ on normal voltage range.

This case will happen when *P* ≈ *P*_load_ by enough to make −24.39% ≤ Δ*P*/*P* ≤ 38.41%. When an islanding condition occurs, *V*_PCC_ will not be less than 187 V and not more than 253 V. The model set the islanding condition at 0.2 s, which the results of the simulation show in Figure 18.

From Figure 18 it can be seen that when the grid was off, islanding condition at 0.2 s, *V*_PCC_ cannot be less than 187 V and not more than 253 V. Then the control system checked occurrence condition by injecting constant voltage (184.8 V (84%) for three cycles) into the load.

If this occurrence condition was an islanding condition, the constant voltage of 184.8 V for three cycles was injected into the load. When the voltage was measured at the PCC, *V*_PCC_ was the same constant voltage 184.8 V for three cycles. Then the control system of the inverter ordered DG off at the point of *V*_PCC_  (rms) < 187 V. In Figure 18 it can be seen that DG ceased to energize the electric power systems network at 0.28 s, showing that the proposed method used times for anti-islanding condition of case of −24.39% ≤ Δ*P*/*P* ≤ 38.41% were within 0.08 s. However, if the occurrence conditions were not an islanding condition, the next case was used.

### 6.4. Case of Injecting a Signal to Change the Undervoltage

This case shows the change when the inverter injected constant voltage (184.8 V (84%) for three cycles) into the load, but the occurrence condition is not an islanding condition. In simulation, set signal of undervoltage shift was injected between 0.4 and 0.46 s.

**Figure 19:**
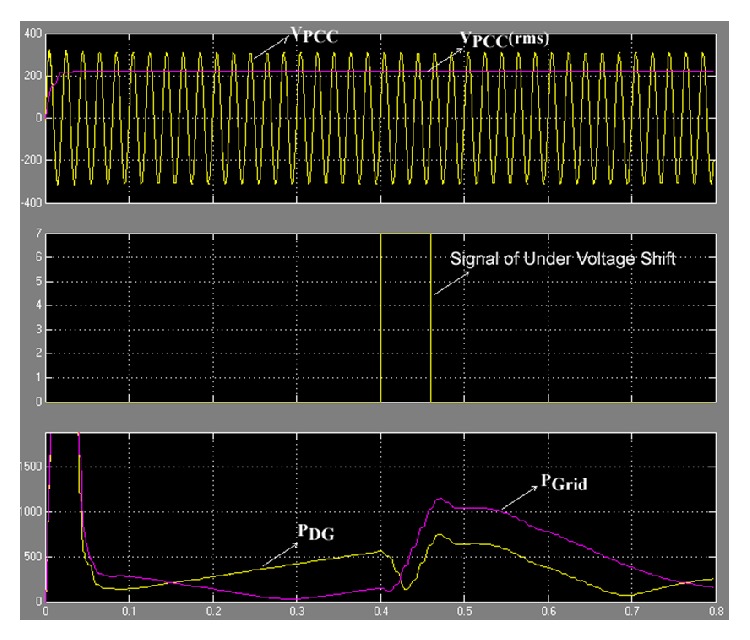
Injecting a signal to change the undervoltage in normal condition.

From Figure 19 it can be seen that *V*_PCC_ did not change, but at the moment the injected signal of undervoltage shift affected reduction of *P*_DG_ and increment of *P*_Grid_, which is consistent with (9).

(10) $$\begin{array}{c} \Delta P_{\text{DG}}+\Delta P_{\text{Grid}}=2\cdot \Delta V\cdot \sqrt{\frac{P_{\text{load}}}{R}}. \end{array}$$

## 7. Conclusion

Simulation results on MATLAB/SIMULINK show that over/undervoltage and undervoltage shift of hybrid islanding detection method is very effective because it can determine anti-islanding condition very fast. Δ*P*/*P* > 38.41% could determine anti-islanding condition within 0.04 s; Δ*P*/*P* < −24.39% could determine anti-islanding condition within 0.04 s; −24.39% ≤ Δ*P*/*P* ≤ 38.41% could determine anti-islanding condition within 0.08 s. This method perturbed the system, only in the case of −24.39% ≤ Δ*P*/*P* ≤ 38.41% at which the control system of inverter injected a signal of undervoltage shift as necessary to check if the occurrence condition was an islanding condition or not.

## Figures and Tables

**Table 1:** Bus records.

Bus	Nom kV	PU Volt	Volt (kV)	Angle (Deg.)	Load MW	Load Mvar	Gen MW	Gen Mvar	Shunts Mvar
1	115	1	115	24.67	21.7	12.7	273.96	29.28	
2	115	0.93744	107.805	18.25	94.2	19			20.56
3	115	0.94066	108.176	23.14	47.8	−3.9			
4	115	0.94857	109.086	23.98	7.6	1			
5	22	0.94857	20.869	23.98	11.2	7.5			21.59
6	22	0.94066	20.694	23.14					
7	69	0.94066	64.905	23.14					16.81
8	22	0.94066	20.694	23.14	29.5	16.6			
9	22	0.93375	20.542	22.92	9	5.8			
10	22	0.93722	20.619	23.28	3.5	1.8			
11	22	0.93237	20.512	22.9	6.1	1.6			
12	22	0.92752	20.405	22.8	13.5	5.8			
13	22	0.91457	20.121	21.71	14.9	5			

**Table 2:** Line and transformer records.

From bus	To bus	Branch device type	Xfrmr	*R*	*X*	*B*
1	2	Line	No	0.047	0.198	0.0025
1	3	Line	No	0.0581	0.0176	0.002
1	4	Line	No	0.0569	0.0174	0.002
2	3	Line	No	0.067	0.171	0.0008
3	4	Line	No	0.0133	0.0421	0
3	6	Transformer	Yes	0	0.00001	0
3	8	Transformer	Yes	0	0.00001	0
4	5	Transformer	Yes	0	0.00001	0
5	10	Line	No	0.095	0.1983	0
5	11	Line	No	0.1219	0.2562	0
5	12	Line	No	0.0661	0.1302	0
6	7	Transformer	Yes	0	0.00001	0
6	8	Transformer	Yes	0	0.00001	0
8	9	Line	No	0.031	0.0847	0
8	13	Line	No	0.126	0.2707	0
9	10	Line	No	0.0826	0.1921	0
11	12	Line	No	0.2211	0.2004	0
12	13	Line	No	0.1715	0.3471	0

**Table 3:** The change of load affecting Δ*P*.

*P*_load_ (MW)	*Q*_load_ (Mvar)	PF	*P*	Δ*P*	*V*_pu_ (%)	Δ*P*/*P* (%)	NDZ
1	0.64	0.84	9	−7.9761	94.92	−88.6233	Off
3	1.92	0.84	9	−5.9845	94.65	−66.4944	Off
5	3.2	0.84	9	−3.9909	94.37	−44.3433	Off
7	4.48	0.84	9	−1.9898	94.1	−22.1089	On
9	5.8	0.84	9	0.0103	93.83	0.1144	On
11	7.04	0.84	9	2.0262	93.55	22.5133	On
13	8.32	0.84	9	4.0322	93.27	44.8022	Off
15	9.6	0.84	9	6.0478	92.99	67.1978	Off
17	10.88	0.84	9	8.0677	92.71	89.6411	Off

**Table 4:** Relationship between voltage and active power of inverter.

*P*_load_ (W)	*P* (W)	Δ*P* (W)	Δ*P*/*P* (%)	On-grid			Off-grid
				*V*_PCC_ (V)	*V*_PCC_ × 115%	*V*_PCC_ × 85%	*V*_PCC_ (V)
600	1,000	−400	−40	226.3	260.2	192.4	253.2
700	1,000	−300	−30	233.4	268.4	198.4	249.3
800	1,000	−200	−20	231.6	266.3	196.9	240.5
900	1,000	−100	−10	229.8	264.3	195.3	191.0
1,000	1,000	0	0	229.8	264.3	195.3	175.0
1,100	1,000	100	10	226.3	260.2	192.4	159.1
1,200	1,000	200	20	229.8	264.3	195.3	148.5
